# Proposal and Validation of a New Classification of Surgical Outcomes after Colorectal Resections within an Enhanced Recovery Programme

**DOI:** 10.1155/2021/8864555

**Published:** 2021-05-11

**Authors:** Giovanni D. Tebala, Waseem Hameed, Salomone Di Saverio, Gaetano Gallo, Giles Bond-Smith

**Affiliations:** ^1^Surgical Emergency Unit, John Radcliffe Hospital, Oxford University NHS Foundation Trust, Oxford, UK; ^2^General Surgery Department, Wexham Park Hospital, Frimley Health NHS Foundation Trust, Slough, UK; ^3^General Surgery Department, University of Insubria, Varese, Italy; ^4^General Surgery Department, Magna Graecia University, Catanzaro, Italy

## Abstract

**Background:**

Advantages of Enhanced Recovery (ER) programmes in colorectal surgery have already been demonstrated, but heterogeneity exists with respect to the choice of compared outcomes. A comprehensive classification aimed at standardizing the reporting of surgical outcomes has been proposed and validated.

**Method:**

Clinical variables of 231 patients who underwent colorectal resections within an ER programme from 2013–2018 were analysed. Their outcomes have been reported according to a new classification in 5 classes and 11 subclasses. Prognostic variables have been identified.

**Results:**

Seventy-nine patients (34.2%) had an optimal class 1 outcome. Almost half of the patients had an uneventful recovery after being discharged after day 4 (2a). Only two patients (0.9%) were discharged early and then readmitted for a minor ailment (2b). Total morbidity was 12.6% (3a–5). Perioperative mortality was 2.6% (5). Young age, laparoscopic resection, and years of experience with ER have been identified as independent prognostic factors towards a totally positive outcome.

**Conclusions:**

The proposed outcome classification is a simple and objective tool to report the surgical outcome in clinical studies. Its implementation seems to be appropriate, in particular, in the field of ER protocols in colorectal surgery, but it can have a wider application in any other surgical subspeciality.

## 1. Introduction

Enhanced Recovery (ER) principles, originally proposed by Kehlet et al. less than 20 years ago [1, 2], are considered the basis of ideal perioperative management of surgical patients today. ER programmes obtained worldwide acceptance, particularly in colorectal surgery where their benefits have already been clearly demonstrated [3–7]. However, ample variability exists within the colorectal literature on the methods of evaluation of surgical outcomes in such patients. Usually, authors tend to consider a whole range of variables from length of postoperative stay to morbidity and mortality, but no attempts have ever been made, to our knowledge, at standardizing the reports of surgical outcomes. In this study, we propose and validate a new classification of surgical outcomes in patients who underwent a colorectal resection within an ER programme.

## 2. Materials and Methods

Clinical data of patients who underwent colorectal resections under an Enhanced Recovery (ER) in Colorectal Surgery Programme from March 2013 to March 2018 at the Colorectal Unit of Noble's Hospital (Isle of Man) were prospectively collected, totally anonymised, and saved into an electronic database (Microsoft Excel for Mac 2011, v.14.7.2). The first Author of this paper was Colorectal Lead of the Noble's Hospital at the time of data collection. Outcomes for these patients have been classified taking into account five variables: length of postoperative stay (within 4 days or more than 4 days), postoperative complications (Clavien–Dindo > 2) [8], unplanned readmission, unplanned reoperation, and mortality within 90 days from the index operation. On the basis of these outcome variables, we have designed the classification system that is reported in Table 1.

Variables analysed in this study have been purposely chosen to be basic, and the proposed classification has been intentionally maintained as simple as possible, for the sake of reproducibility.

Patients who were discharged within day 4 and did not have any readmission or complication or reoperation or any 90-day mortality were considered to have obtained the optimal results (Class 1); therefore, this has been considered the “index” outcome for future comparisons. Patients who were discharged later on day 4 and/or were readmitted, but without serious complications, belong to class 2. Patients who had Clavien–Dindo > 2 complications but did not need a reoperation were classified as class 3, whereas those who needed a reoperation belong to class 4. Class 5 is for patients who died from a surgical complication within 90 days from the index operation.

Data have been analysed with a specific statistical package (IBM SPSS v.22.0).

Potentially causative variables have been identified with univariate analysis and multivariate analysis. Comparison of frequencies between groups for ordinal or nominal variables has been performed with Pearson's chi-square test. All the possible prognostic variables that reached statistical significance at univariate analysis were subsequently entered into a logistic regression analysis (multivariate). Odds ratios were calculated as the exponential of the coefficient in the regression model.

The details of our Enhanced Recovery Programme have been reported and discussed elsewhere [6, 9].

All the patients involved in this study gave informed consent to the operation and any other subsequent procedure. Formal approval of this study by the Local Research Ethical Committee was not considered to be necessary due to the retrospective nature of the study. Formal written consent to participate in the study was not obtained from participants because the study reports the results of a retrospective analysis of anonymised clinical data.

## 3. Results

In the period of the study, 231 patients underwent colorectal resections. There were 122 males and 109 females, with a mean age of 69.1 ± 11.7 years. The distribution of the prognostic factors is reported in Table 2.

**Figure 1:**
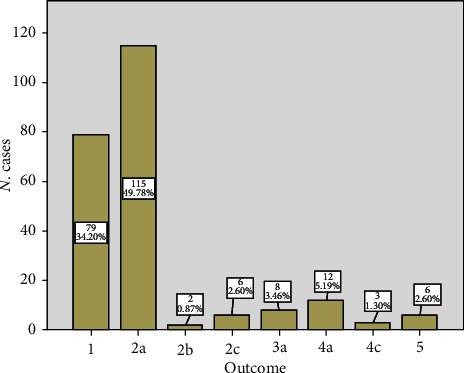
Distribution of outcomes. No patients for classes 3b, 3c, and 4b.

Distribution of the recorded outcomes is reported in Figure 1.

Seventy-nine patients (34.2%) had an optimal outcome, class 1, whereas 152 patients (65.8%) were considered to have a suboptimal outcome. Only two patients (0.9%) were discharged early and then readmitted, but none of them had a Clavien–Dindo > 2 complication (class 2b). No patients were readmitted after early or late discharge with significant complications (3b or 3c) or for an unplanned reoperation (4b). Almost half of the patients had an uneventful recovery after being discharged following day 4 (2a). Total morbidity was, therefore, 12.6% (29 patients, class 3a to 5). Perioperative mortality was 2.6% (6 patients, class 5).

At univariate analysis age < 65, malignant disease, elective operation, laparoscopic approach, and years since adoption of the ER protocol were identified as factors associated with a positive outcome. However, multivariable analysis (Table 3) only confirmed young age, laparoscopic resection, and years of experience with ER as independent factors towards a totally positive outcome.

Gender, surgery site, and experience of the surgeon did not appear to influence the results.

## 4. Discussion

ER programmes are an essential part of the current management of patients undergoing colorectal surgery. Several Authors have estimated their impact on the surgical results, and their multiple advantages in terms of reduced morbidity and length of stay have been widely proven [9, 10].

However, heterogeneity still exists on the primary endpoints of outcome studies. In fact, most authors consider as their primary endpoints length of postoperative stay (LOS), unplanned readmission rate (RAR), morbidity, unplanned reoperation rate (ROR), and mortality [11]. Others also include patient-reported outcomes [12] or the Abdominal Surgery Impact Scale [13] that measures the quality of life after abdominal surgery according to patient-reported parameters.

Clearly, the former are far more objective and standardizable, whereas patient-reported outcomes are often quite subjective and do not fully estimate the efficacy of surgery.

In a meta-analysis by Messenger et al., it was reported that 70 studies described 159 different outcome measures and that some of them were poorly defined [14].

We decided to build up a classification system based on four empirically chosen and clearly defined dependent variables (LOS, RAR, morbidity, ROR, and mortality).

The decision to consider LOS and not total length of stay was based on the consideration that preoperative stay might be influenced by organization factors not necessarily dependent on the clinical status of the patient. To further standardize the results, we empirically chose a cutoff at 4 days, considering that, ideally, a noncomplicated patient undergoing colorectal resection under an ER programme should leave hospital within 4 days, as reported by a 2017 study by the PeriOperative Italian Society [7]. However, we appreciate that. in some cases, a very early discharge may not be possible due to specific factors (lack of family/community support, stoma care, and administrative issues) not related to any complication. For this reason, the group of “optimal” results (class 1) is followed by an almost equally positive results group (class 2a), whose patients have been discharged a little later but with no significant complication.

The risk of unplanned readmission and/or reoperation due to a complication happening after discharge has always been a great concern for those who, at least initially, opposed the ER principles. Several studies demonstrated that the risk of complications is not correlated to LOS and that patients discharged early have a generally better outcome with respect to those discharged later [4, 15]. As stated elsewhere, in the absence of a reliable randomized trial, it is not completely clear if sending patients home earlier reduces the risk of complications by avoiding hospital-acquired infections and issues associated with prolonged stay in bed or if patients that are discharged early are those who have a straightforward postoperative recovery and, therefore, would not have suffered complications anyway [9]. We have also already demonstrated [9], together with other authors [3], that early discharge is not associated with increased risk of unplanned readmission.

Perioperative morbidity and mortality are the main endpoints of all the surgical studies and have, therefore, been added to our classification. We decided to consider mortality and morbidity within 90 days from surgery instead of 30 days, to also include late complications and side effects [16].

While the majority of available studies reported all these variables as primary or secondary outcomes, no comprehensive classification combining multiple variables in a single scheme has ever been reported, to our knowledge.

We validated this new classification with our series of patients undergoing bowel resection within an ER programme.

The focus of our evaluation was not colorectal surgery in its wider meaning, but the impact of the ER programme on patients undergoing colorectal surgery. Therefore, we considered results of all the patients in class 2a and above as “suboptimal,” whereas those on class 1 were supposed to have had the best possible outcome of an uneventful surgical operation associated with early discharge and fast recovery, according to the principles of ER. Similarly, Oh et al. considered successful ER to be when patients were discharged within day 5 with no complications [17].

Clearly, other researchers or clinicians can decide to group their outcomes in different ways, wish to analyze the outcomes of general colorectal surgery, irrespective of an ER programme, group together class 1 and 2a (noncomplicated and nonreadmitted), or class1, 2a, 2b, and 2c (noncomplicated patients), and use them as control groups with respect to patients with suboptimal results (classes 3 to 5).

In our series, 34.2% of patients were classified as class 1. This rate represents not only patients who were discharged early but also those who were discharged early and were not readmitted or reoperated and whose clinical course was uneventful, that is, patients who were thought to have obtained the maximum advantage from the ER protocol. If we add patients with class 2a results, the rate of positive outcomes goes to 84%. Unfortunately, these data cannot be compared with other studies yet, but the adoption of the proposed classification would hopefully provide a common language to evaluate and compare the results of ER.

Presenting and discussing our results is not the primary scope of this work; however, it is worth confirming that once again the laparoscopic approach proved to be a crucial component of ER programmes with both univariate and multivariate analysis. In fact, almost half of the patients who had laparoscopic surgery had class 1 outcomes, thus confirming that minimally invasive approaches are also able to reduce the risk of complications, readmissions, and reoperations and not just LOS. It is our opinion that the full potential of ER principles can be obtained not only in elective patients undergoing surgery by laparoscopic approaches but also in emergency patients and those operated using open approaches, although the lack of a thorough preoperative optimization in emergency patients and the issue of pain control in patients undergoing open surgery can hamper the overall results. In our series, only 8% of patients operated on by open surgery had class 1 outcome. For obvious reasons, young age and years of experience with the ER protocols are also independently associated with good results.

Experience of the surgeon did not influence the results. This can probably be explained by the observation that, in our series, nonexperienced surgeons are always supervised by an experienced consultant during cancer resections.

The main advantage of this classification is that vastly reduces subjectivity. In fact, the considered parameters are obvious and easily measurable. The choice to consider only Clavien–Dindo > 2 complications (that is, need for active invasive treatment of a complication) is to eliminate the only possible unclear point and the discretion potentially associated with Clavien–Dindo grades 1 and 2.

In conclusion, we believe that the proposed outcome classification is a simple, objective, and easy-to-standardize tool to report and compare surgical outcomes in different studies or the various prognostic factors within the same study. Its implementation seems to be appropriate, in particular, in the field of ER protocols in colorectal surgery, but it can have a wider application in any other surgical subspeciality. Although our validation clearly demonstrates the reliability of the classification, we appreciate it may need to be externally revalidated on a bigger sample.

## Figures and Tables

**Table 1:** Classification of surgical outcomes after colorectal resection.

Class	Discharge after day4	Unplanned readmission	Postop complications (Clavien–Dindo > 2)	Unplanned reoperation	Mortality
1	No	No	No	No	No
2a	Yes	No	No	No	No
2b	No	Yes	No	No	No
2c	Yes	Yes	No	No	No
3a	Yes	No	Yes	No	No
3b	No	Yes	Yes	No	No
3c	Yes	Yes	Yes	No	No
4a	Yes	No	Yes	Yes	No
4b	No	Yes	Yes	Yes	No
4c	Yes	Yes	Yes	Yes	No
5	Any	Any	Any	Any	Yes

**Table 2:** Univariable analysis.

Factor	*N* (%)	Outcome 1	Outcome 2a–5	*p*
Total	231	79 (34.2%)	152 (65.8%)	
Gender M	122 (52.8%)	41 (33.6%)	81 (66.4%)	0.841
Gender F	109 (47.2%)	38 (34.9%)	71 (65.1%)	

Age < 65	78 (33.8%)	36 (46.2%)	42 (53.8%)	0.006
Age > 65	153 (66.2%)	43 (28.1%)	110 (71.9%)	

Elective	191 (82.7%)	74 (38.7%)	117 (61.3%)	0.001
Urgent	40 (17.3%)	5 (12.5%)	35 (87.5%)	

Malignant	176 (76.2%)	70 (39.8%)	106 (60.2%)	0.001
Nonmalignant	55 (23.8%)	9 (16.4%)	46 (83.6%)	

Right colon*∗*	84 (36.4%)	33 (39.3%)	51 (60.7%)	0.424
Left colon*∗∗*	70 (30.3%)	25 (35.7%)	45 (64.3%)	
Rectum	68 (29.4%)	19 (27.9%)	49 (72.1%)	
Total colect.§	9 (3.9%)	2 (22.2%)	7 (77.8%)	

Experienced surgeon	217 (93.9%)	75 (34.6%)	142 (65.4%)	0.647
Nonexperienced surgeon&	14 (6.1%)	4 (28.6%)	10 (71.4%)	

Laparoscopic	148 (64.1%)	72 (48.6%)	76 (51.4%)	0.000
Open $	83 (35.9%)	7 (8.4%)	76 (91.6%)	

Year 1	37 (16.0%)	5 (13.5%)	32 (86.5%)	0.010
Year 2	53 (22.9%)	16 (30.2%)	37 (69.8%)	
Year 3	43 (18.6%)	16 (37.2%)	27 (62.8%)	
Year 4	50 (21.6%)	25 (50.0%)	25 (50.0%)	
Year 5	48 (20.8%)	17 (35.4%)	31 (64.4%)	

*∗*Including transverse and splenic flexure; *∗∗*including sigmoid; §, including subtotal colectomies; &, supervised by an experienced consultant; $, including lap to open conversions.

**Table 3:** Multivariable analysis.

	Coefficient	Odds ratio	*p*
Laparoscopic resection	2.505	12.248	0.000
Age < 65 yrs	0.887	2.427	0.008
Year	0.386	1.471	0.002
Constant	-4.044		0.018

Dependent variable: outcome 1 (vs. 2a–5).

## Data Availability

The datasets used and/or analysed during the current study are available from the corresponding author on reasonable request, within the limitations of the Isle of Man regulations on confidentiality.
